# Direct Outreach in Bars and Clubs to Enroll Cigarette Smokers in Mobile Cessation Services: Exploratory Study

**DOI:** 10.2196/28059

**Published:** 2022-06-02

**Authors:** Patricia Chalela, Alfred L McAlister, Cliff Despres, Edgar Muñoz, Pramod Sukumaran, David Akopian, Sahak Kaghyan, Jesus Trujillo, Amelie G Ramirez

**Affiliations:** 1 Institute for Health Promotion Research University of Texas Health Science Center at San Antonio San Antonio, TX United States; 2 School of Public Health Austin Regional Campus University of Texas Health Science Center at Houston Austin, TX United States; 3 College of Engineering The University of Texas at San Antonio San Antonio, TX United States; 4 Foundry512 Austin, TX United States

**Keywords:** smoking cessation, young adults, Latinos, mobile intervention, direct recruitment

## Abstract

**Background:**

Cigarette smoking and alcohol use are well known to be concomitant behaviors, but there is a lack of studies related to recruitment of smokers for mobile cessation services at places where alcohol is consumed, such as bars and clubs. Adapting recruitment strategies to expand the reach of cessation programs to where tobacco users are located may help decrease the health-equity gap in tobacco control by improving reach and enrollment of underserved smokers residing in low-income and rural areas who are not reached by traditional cessation services.

**Objective:**

The purpose of this exploratory study was to assess the feasibility of direct outreach in bars, clubs, and restaurants to recruit smokers to Quitxt, our mobile smoking cessation service. Quitxt is delivered through SMS text messaging or Facebook Messenger.

**Methods:**

We collaborated with an advertising agency to conduct in-person recruitment of young adult smokers aged 18-29 years, focusing on urban and rural Spanish-speaking Latino participants, as well as English-speaking rural White and African American participants. Street team members were recruited and trained in a 4-hour session, including a brief introduction to the public health impacts of cigarette smoking and the aims of the project. The street teams made direct, face-to-face contact with smokers in and near smoking areas at 25 bars, clubs, and other venues frequented by young smokers in urban San Antonio and nearby rural areas.

**Results:**

The 3923 interactions by the street teams produced 335 (8.5%) program enrollments. Most participants were English speakers with a mean age of 29.2 (SD 10.6) years and smoked a mean of 8.5 (SD 6.2) cigarettes per day. Among users who responded to questions on gender and ethnicity, 66% (70/106) were women and 56% (60/107) were Hispanic/Latino. Among users ready to make a quit attempt, 22% (17/77) reported 1 tobacco-free day and 16% (10/62) reported maintaining cessation to achieve 1 week without smoking. The response rate to later follow-up questions was low.

**Conclusions:**

Direct outreach in bars and clubs is a useful method for connecting young adult cigarette smokers with mobile cessation services. However, further research is needed to learn more about how mobile services can influence long-term smoking cessation among those recruited through direct outreach, as well as to test the use of incentives in obtaining more useful response rates.

## Introduction

Quitxt (Institute for Health Promotion Research) is a mobile service for smoking cessation designed to guide users through the process of quitting and maintaining abstinence from tobacco use. Quitxt is available via SMS text messaging and Facebook messaging, in both English and Spanish, with enrollment promoted mainly through social media advertising. The program does not offer incentives for responding to assessment questions. Prior studies have demonstrated that Quitxt can be effective in helping up to 1 in 5 users to stop smoking for up to 6 months [1,2]. In this study, we sought to recruit users directly at locations where smokers are frequently encountered: in bars and clubs where alcohol is consumed.

Previous research has found that social media can reach young adults wherever they are, at any time of the day [3]. With the potential for private interactions and public peer outreach, Facebook is a useful, cost-effective recruitment source for young adult smokers [1,4,5]. Mobile devices also have great potential to provide personalized smoking cessation support services, as a Cochrane Review [6] has concluded that text message–based interventions can significantly increase odds of successful cessation of smoking. Many programs have demonstrated that an outreach program using workshops to deliver smoking cessation treatment is effective and easily adoptable by existing public health organizations [7]. Adapting recruitment strategies to expand the program by reaching tobacco users where they are located may help decrease the health-equity gap in tobacco control by improving reach and enrollment of underserved smokers residing in low-income and rural areas who are not reached by traditional cessation services [8].

Despite much research on innovation in cessation services, there were comparatively few reports on methods other than media promotion and advertising for recruiting participants into such services. In a widely cited study, Lando et al [9] reported success from telephone outreach to numbers listed in defined populations for recruiting smokers into telephone counseling. McClure et al [10] found that proactive invitation letters to newsletter-recruited smokers could enhance enrollment in an internet-based service. “Targeting” potential enrollees via direct face-to-face outreach was recommended, but not reported, by Chevalking et al [11] for recruitment to mobile apps similar to the one used in this study. In fact, direct outreach to tobacco users where they can most easily be found is increasingly recognized as an important way to recruit them to cessation services [12-15]. Although internet communication is easy to organize and may be highly cost-effective, person-to-person recruitment to directly reach potential participants in person may be the best way to reach those with lower incomes, lower education levels, and difficulties accessing the internet [13]. Recently published reports described the deployment of “street teams” to reach teens and young adults through peer-to-peer outreach strategies at community venues and events [16]. Outreach at places of employment has been reported for underserved Latino communities at urban construction sites [12]. A very promising approach has been reported from Hong Kong, in which tobacco users are found and recruited directly at outdoor “smoking hotspots” [16-18]. Other very recent research described positive results from the distribution of free nicotine replacement products as a part of direct outreach at smoking hotspots [19-23]. Cigarette smoking and alcohol use are well known to be concomitant behaviors. Most smokers drink alcohol, smokers are more likely to drink than nonsmokers, and those who use both may use them together in the same situations [24,25]. In addition, alcohol consumption is associated with smoking persistence, smoking relapse, and lower odds of quitting success [26-28]. However, there is a lack of research on smokers’ recruitment for mobile cessation services at places where alcohol is consumed, such as bars and clubs. This represents an approach that clearly deserves exploration of feasibility, and thus is the subject of this study.

To explore the feasibility of direct recruitment at venues where alcohol is consumed, we collaborated with an advertising agency (Foundry512) that deployed street teams of outreach workers to recruit smokers to join our Quitxt program at bars, clubs, and other venues in and near San Antonio, Texas. Direct recruitment was done during a brief interval before the COVID-19 pandemic, which made such face-to-face work in bars and clubs unfeasible.

## Methods

### Ethics Consideration

This study did not require Institutional Review Board approval because it is not a regulated research as defined by the Department of Health and Human Sciences (DHHS) regulations at 45 Code of Federal Regulations (CFR) 46 and FDA regulations at 21 CFR 56 [29].

The proposed program is not funded as research and is not a systematic investigation to test a hypothesis and permit conclusions to be drawn. Further, the purpose is not to investigate the safety or effectiveness of a drug, medical device or biologic.

### Target Audience and Recruitment

The target audience for this work was individuals aged 18-29 years, and we focused on urban and rural Spanish-speaking Latino smokers, as well as English-speaking rural White and African American smokers. The face-to-face recruitment was carried out by Foundry512, a full-service advertising agency with extensive experience in the promotion of alcohol products in places where alcohol is consumed.

### Selecting and Training Street Team Members

Foundry512 hired and trained 9 part-time workers to form “street teams.” To help ensure street team members could establish a positive relationship with smokers, selection was done to ensure that the team mirrored the culture and demographics of our target audiences. Of the team members, most were bilingual, all had previous experience in outreach marketing, and they were either former smokers or had smokers in their social circles.

Street teams were trained to use a direct-interaction approach to engage users who were observed using tobacco. The main training objectives were to raise awareness and empathy for the subject and to prepare the teams to follow the program’s methodology. The training was conducted in 2 sessions that lasted 2 and 4 hours, respectively. During the first session, street team members received information about smoking, risk factors, side effects, and how the advertising industry has influenced smoking. In addition, the elements that contribute to successful in-person interactions with someone who tries to quit or is thinking about quitting were explained. The second session included a brief introduction to the public health impacts of cigarette smoking and the aims of the Quitxt program and its mobile platforms. Lastly, the training focused on preparing the teams for questions or doubts that smokers may have during the interaction, using empathetic approaches and responses that reflect the challenge of quitting, and learning ways to handle difficult situations or extremely negative responses. Both modeling demonstrations and role playing were used to help the street teams learn the guidelines for interaction, feel more comfortable with the questions to be asked, and make their interactions flow naturally and empathetically.

### Approaching Potential Participants

Foundry512 identified and selected 25 bars, clubs, and other venues and events frequented by young adult smokers in urban San Antonio and nearby rural areas. They developed a daily schedule and assigned street team members to different venues and events. When smokers were encountered, street team members guided the interaction using three short questions designed to obtain information about the intention or motivation that smokers had to quit smoking: (1) How important is it to you to quit smoking? (2) How confident are you in your ability to quit smoking? and (3) How ready do you feel to quit smoking? Response options used a rating scale of 1-10 to assess the smokers’ level of motivation and readiness to quit smoking. This schema was designed by Foundry512 to be used as a guide to create fluid conversation and to manage complicated interactions with smokers who are apathetic or conversations that would exceed time limits.

The street teams made face-to-face contact with smokers at selected locations and conducted interactions that had an average duration of 4.5 minutes each. In the interactions, street teams first established rapport with the smokers and then toward the end of the engagement introduced the Quitxt mobile cessation program, explained its functions and benefits, and invited them to join the program.

### The Quitxt Mobile Cessation Program

Quitxt is a bilingual, evidence-based mobile cessation service that is free to use and culturally appropriate. It turns the participants’ phones into a personal coach to help young adults quit smoking. To enroll in the program, participants should be 18 years of age or older, current smokers, and willing to set a quit date within 14 days and provide baseline data [2]. The Quitxt SMS text messaging and Facebook Messenger platforms each contain a complex sequence of interactive messaging, beginning with collection of baseline data that include basic demographics (ie, age, ethnicity, gender), number of cigarettes smoked per day, and e-cigarette use. Participants are then prompted to either choose “quit tomorrow” or set a “quit date” within 2 weeks. Based on the selection, a specific sequence of messages follows. The program provides motivational messages, tips to manage cravings and difficult situations, and 24/7 support. After their quit date, enrollees are also encouraged to text “help” if they are having difficulty avoiding cigarettes. When they text “help,” the system texts to ask if the help needed is due to “stress” or “mood”; depending on their text reply, they are then sent either a link to breathing exercises (for stress) or a message with links to diverting, humorous videos (for mood) [2].

### Data Analysis

To describe the characteristics of participants referred by the street teams and enrolled in the program, categorical and continuous variables were summarized with frequency distribution, cross-tabulation, mean, and standard deviation as appropriate. Cessation rates were calculated as the proportion of users who reported abstinence among the total active users at the time of the assessment, which were completed at 1, 7, 28, and 72 days from the quit date set by each smoker [30].

## Results

The street teams reported 3923 interactions and 353 enrollments of smokers who fit the target group. According to street team members, the most useful question they asked was “How important is it to you to quit smoking?” The least useful was “How ready do you feel to quit smoking?” Street team members stated that this question generated negative reactions and refusals. Therefore, based on the team members’ assessments, Foundry512 decided to take out this question with the purpose of maximizing positive reactions from smokers. Interestingly, street team members reported that Latino smokers did not use Spanish as their primary language in these types of social settings. It is also notable that suspicion of the street teams’ legitimacy and lack of receptivity were overcome by designing and providing a badge and lanyard for the street teams to look like a cohesive unit with a unified purpose.

Although 353 enrollments were reported by the street teams, data were obtained from 335 users who formally enrolled in the Quitxt SMS (n=317) and Quitxt Facebook Messenger (n=18) services (Table 1).

All but 5 participants preferred the protocol in English. Only 114 participants provided their age (mean 29.2, SD 10.6 years). Of the 106 participants who reported their gender, 66% (n=70) were female. Ethnicity was reported by 107 enrollees and 56% (n=60) identified themselves as Hispanic/Latino. Among the 80 who reported their current tobacco use rate, the mean consumption was 8.5 (SD 6.2) cigarettes per day.

Among these 335 formally enrolled participants, 77 (23%) reported that they were ready to make a quit attempt within 2 weeks. The protocol included questions about smoking cessation during the process of quitting. Among users who were ready to make a quit attempt, 22% (17/77) reported abstinence at quit day 1, and 16% (10/62) reported maintaining cessation to achieve 1 week without tobacco use. Response rates to later follow-up queries were very low. Only 2 responded to the query about tobacco use 28 days after their quit attempt, and 1 of those reported maintained cessation. There were 3 participants who responded to a similar question 72 days after their quit attempt, and all 3 of these reported that cessation had been maintained for that interval.

**Table 1:** Characteristics of participants (N=335) recruited by street teams.

Characteristics			Value^a^
**Preferred language (N=335), n (%)**			
	English	330 (98)	
	Spanish	5 (2)	
Age (years; n=114), mean (SD)			29.2 (11)
**Gender (n=106), n (%)**			
	Male	36 (34)	
	Female	70 (66)	
**Ethnicity (n=107), n (%)**			
	Hispanic/Latino	60 (56)	
	Non-Hispanic/Latino	47 (44)	
Cigarettes smoked/day (n=80), mean (SD)			8.5 (6)
Ready to make a quit attempt (N=335), n (%)			77 (23)
Cessation at quit day 1 (n=77)			17 (22)
Cessation at quit day 7 (n=62)			10 (16)

^a^Percentages were calculated based on responses received, as some participants did not respond to or skipped over questions.

## Discussion

### Principal Findings

Our results suggested that face-to-face recruitment of young adult smokers at venues where alcohol is served (ie, clubs, bars, restaurants) was a feasible strategy to enroll both English- and Spanish-speaking young adults into a mobile cessation service. As in the studies conducted by Asfar et al [12], Chan et al [15], and Saw et al [16], the Quitxt street teams directly recruited participants at places that are frequented by young adult smokers; however, none of their studies involved a mobile phone cessation program. Given that young adults are less likely to seek or use traditional cessation services, such as quitlines [2], direct outreach to young adult smokers by street teams of peers may improve reach and enrollment to smoking cessation services, particularly if the program is tailored to the participants’ culture, language, and media use.

There are some limitations to consider. This was not a randomized controlled study and we were unable to assess the effectiveness of the street teams’ approach compared with social media advertising, which is the most common strategy used by Quitxt to promote and recruit participants into the program. The fidelity of the intervention was not assessed, and the street team members’ adherence to its methodology was unclear. As has happened to many programs across the country, the COVID-19 pandemic impacted our program activities. Face-to-face recruitment by Foundry512 had to be canceled 2 weeks before completing the scheduled activities due to shutdown of bars, restaurants, clubs, and public events to protect the public and avoid the spread of the virus. Even though COVID-19 cut short the face-to-face recruitment efforts, the street teams’ approach showed promising results regarding its feasibility in recruiting for a mobile cessation service.

There were lessons learned and challenges experienced during the implementation. First, selecting street team members who share similar demographic characteristics as the target groups is important and facilitates interaction and engagement. Second, implementing hands-on training that incorporates role play is essential to building team confidence. Third, having formal badges, business cards, or other forms of formal identification for street teams provides legitimacy, generates trust, and facilitates interactions with potential participants. The initial lack of standardized identification during the first activation caused people to be less receptive to the street teams, which led potential participants to question the legitimacy of the activity. Foundry512 resolved this issue by designing and providing a badge and lanyard that included the team member’s name and the Quitxt and University of Texas Health Science Center at San Antonio logos. In this way, the street team members looked like a formal cohesive unit with a unified purpose. Fourth, having a contingency plan is important, as well as verifying representation of the target groups in the venues selected during planning. During the first activation, the target audiences were not found at the scheduled locations. Additional research was carried out by Foundry512 as part of a contingency plan and new venues were successfully identified by the team. Lastly, maintaining close communication and creating opportunities for additional training is crucial for adapting the intervention to unexpected situations. The main challenge faced during implementation was the COVID-19 outbreak, which caused both the participants and the street teams to express feelings of concern and vulnerability. As the situation developed day by day, Foundry512 adapted the street team methodology to meet rising concerns and implement safety measures recommended by the local, state, and federal governments. Team members were trained on safety protocols to keep interactions with participants safe (ie, social distancing, wearing masks). Starting with the second activation, street team members were provided with bilingual enrollment cards that included a QR code and 3-step instructions on how to enroll in Quitxt. This provided a safe way to give instructions to interested young smokers, while avoiding close contact with the person or their cell phones.

### Conclusion

Findings from this exploratory study showed that direct outreach in bars and clubs is a feasible method and has great potential for enrolling participants into a mobile smoking cessation program. Nearly 4000 tobacco users were approached and 335 (8.5%) formally registered as enrollees in the program. Among these individuals, approximately 1 in 5 were ready to make a quit attempt and approximately 1 in 7 achieved 1 week of nonsmoking. However, subsequent response rates to assessment questions were too low to draw any conclusions about long-term impact on the users’ smoking status. We concluded that direct outreach in bars and clubs is a useful method for connecting young adult cigarette smokers with mobile cessation services and the use of incentives could help obtain more useful response rates. However, further research is needed to learn more about how mobile services can influence long-term smoking cessation for users recruited through face-to-face interactions.

## Abbreviations

DHHS: Department of Health and Human Sciences
CFR: Code of Federal Regulations
